# Identification of proteins binding coding and non-coding human RNAs using protein microarrays

**DOI:** 10.1186/1471-2164-13-633

**Published:** 2012-11-16

**Authors:** Zurab Siprashvili, Dan E Webster, Markus Kretz, Danielle Johnston, John L Rinn, Howard Y Chang, Paul A Khavari

**Affiliations:** 1 The Program in Epithelial Biology, Stanford University School of Medicine, 269 Campus Drive, Room 2145, Stanford, CA 94305, USA; 2 Department of Stem Cell and Regenerative Biology, Harvard University, Cambridge, MA 02138, USA; 3 Howard Hughes Medical Institute, Stanford, CA 94305, USA; 4 Veterans Affairs Palo Alto Healthcare System, Palo Alto, CA 94304

**Keywords:** Non-coding RNA, Microarray, p53, Ras, Staufen

## Abstract

**Background:**

The regulation and function of mammalian RNAs has been increasingly appreciated to operate via RNA-protein interactions. With the recent discovery of thousands of novel human RNA molecules by high-throughput RNA sequencing, efficient methods to uncover RNA-protein interactions are urgently required. Existing methods to study proteins associated with a given RNA are laborious and require substantial amounts of cell-derived starting material. To overcome these limitations, we have developed a rapid and large-scale approach to characterize binding of in vitro transcribed labeled RNA to ~9,400 human recombinant proteins spotted on protein microarrays.

**Results:**

We have optimized methodology to probe human protein microarrays with full-length RNA molecules and have identified 137 RNA-protein interactions specific for 10 coding and non-coding RNAs. Those proteins showed strong enrichment for common human RNA binding domains such as RRM, RBD, as well as K homology and CCCH type zinc finger motifs. Previously unknown RNA-protein interactions were discovered using this technique, and these interactions were biochemically verified between *TP53* mRNA and Staufen1 protein as well as between *HRAS* mRNA and CNBP protein. Functional characterization of the interaction between Staufen 1 protein and *TP53* mRNA revealed a novel role for Staufen 1 in preserving *TP53* RNA stability.

**Conclusions:**

Our approach demonstrates a scalable methodology, allowing rapid and efficient identification of novel human RNA-protein interactions using RNA hybridization to human protein microarrays. Biochemical validation of newly identified interactions between *TP53*-Stau1 and *HRAS*-CNBP using reciprocal pull-down experiments, both in vitro and in vivo, demonstrates the utility of this approach to study uncharacterized RNA-protein interactions.

## Background

Functional roles for both coding and non-coding RNA molecules have been increasingly appreciated in a variety of biologic processes, including gene regulation, molecular trafficking, and protein translation [1-4]. For example, mRNAs of coding genes are increasingly recognized as targets of translational regulation by a variety of mechanisms [5,6]. Additionally, thousands of long non-coding RNAs have recently been identified [7-9] and a growing number of these are being assigned discrete biologic functions [10-13]. Strategies to identify multiple RNAs binding an individual protein of interest have advanced further than those designed to identify multiple proteins binding a given RNA. Examples of the former include immunoprecipitation followed by microarray hybridization (RIP-Chip) or sequencing (RIP-Seq) [8,14,15]. Available approaches to accomplish the latter include RNA pull-downs in which proteins bound to biotin-labeled RNA are isolated and analyzed by techniques including mass spectrometry [16]. The capacity to rapidly identify multiple proteins binding to an individual RNA of interest will allow characterization of the molecular mechanisms of action and the functional role of specific RNAs in human disease.

The recent use of protein microarrays in yeast [17,18] suggests a similar approach may be of utility in the more complex human setting. Commercially available human protein microarrays were designed for detection of protein-protein interactions using protein or small molecule probes. As protein microarrays permit high throughput screening for intermolecular interactions, they may also provide an alternative approach to study human protein-RNA interactions, an application in which they have not yet been reported. Here, we demonstrate a high-throughput methodology allowing rapid identification of the proteins binding to a given human RNA molecule using a protein microarray containing ~9,400 human recombinant proteins spotted in duplicate. As a result of this approach we identify previously uncharacterized interactions between *HRAS* RNA and CNBP protein, as well as human Stau1 and *TP53* RNA and show that Stau1 influences *TP53* RNA stability in the context of transcriptional blockade.

## Results and discussion

**Figure 1:**
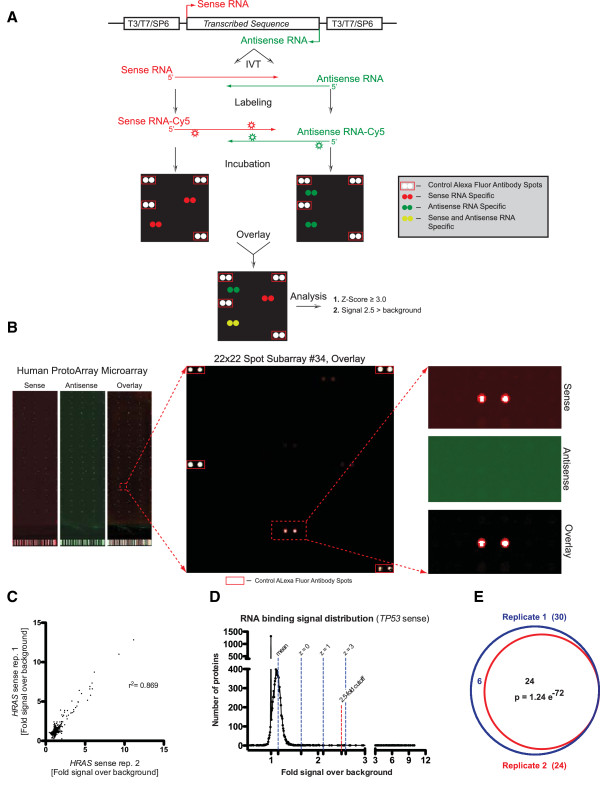
**RNA incubation on human protein microarrays.** (**A**) Experimental and analytical workflow. (**B**) RNA incubation signal on protein microarray. Sense [red] and antisense [green] strand signal is shown for the *SOX2OT* non-coding RNA, with pseudocolor images of independently probed arrays. Panels at left show the entire microarray spotted with ~9400 recombinant human proteins; the middle panel is an enlarged 484 protein spot sub-array and the right panels represent an enlargement of the binding signal demonstrating strand-specific binding to the RBPMS protein [GenBank:BC003608] [all proteins spotted in duplicate; sub-array positive controls boxed in red]. (**C**) Scatter plot of signal intensity above background for all proteins between two independent replicates of the *HRAS* sense mRNA. Pearson correlation r^2^ value is shown at right. (**D**) RNA binding signal intensity over background distribution for *TP53* sense RNA to the all proteins. Mean and Z-Scores (standard deviations from mean) are depicted in blue with selected fold change cutoff of 2.5 in red. A Z-Score ≥ 3 and signal intensity over background ≥ 2.5 are used to select significant RNA-protein binding event. (**E**) Venn diagram of significant hits from two independent *HRAS* mRNA incubations to protein array from (**C**), (p value, Fisher’s exact test).

To define RNA-protein interactions, we utilized sense and antisense strands for 10 RNA transcripts representing protein coding RNAs *TP53, HRAS, MYC, BCL2* and non-coding RNAs *PWRN1, SOX2OT, OCC1, IGF2RNC, lncRBM26* and *DLEU1.* The schematic diagram of the workflow used in this work is presented on Figure 1A and a detailed protocol of probe preparation, labeling and hybridization conditions are included in experimental procedures section as well as in Additional file 1: Figure S1 and Additional file 2: Table S1. Briefly, the aforementioned RNAs were in vitro transcribed, labeled with Cy5 and independently probed on human protein microarrays. The labeling process was optimized in order to achieve ~ 3 pmol dye per μg RNA with an average efficacy of 1 dye molecule for approximately every 850 bp RNA (Additional file 2: Table S1) to minimally influence RNA native structure while yielding signal intensities that are readily visualized (Figure 1B).

In order to determine the reproducibility of RNA-protein interactions observed with this technology, we performed technical replicates of *HRAS* sense RNA incubation to the microarray. Comparison of the ratio of signal intensity above background of every spotted protein between the two independent replicates demonstrated suitable results with a Pearson correlation of r^2^=0.869 (Figure 1C). We next analyzed the distribution of the signal within each array to establish filtering criterion for RNA-protein interaction significance. A representative distribution of signal intensities for all proteins from the *TP53* sense RNA array displaying deviations from the global mean (Z-Scores) is shown on Figure 1D. It is notable that there is a large fraction of proteins that display a signal intensity ratio over background of 1, suggesting the absence of a global non-specific fluorescence. We selected filters allowing identification of the significant RNA-protein binding events based on a Z-Score ≥ 3.0 and a minimum signal above background of 2.5 fold. Gene lists of all significant hits from all hybridized RNAs in this study were generated (Additional file 3: Table S2), and overlaps of hits from the two technical replicates of *HRAS* sense RNA probed to protein array resulted in a significance of p < 10^-72^ (Figure 1E).

**Figure 2:**
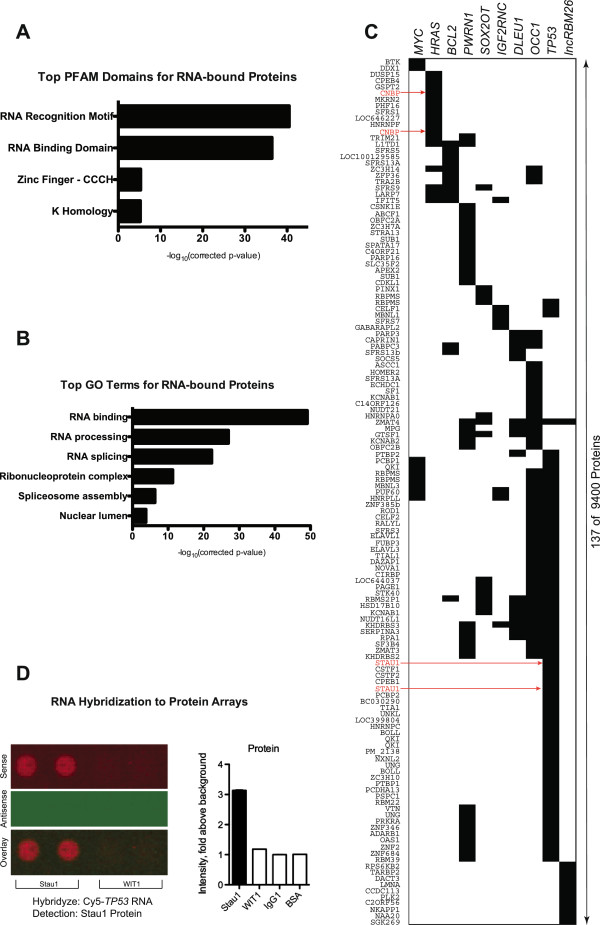
**RNA binding proteins identified by incubation of labeled RNAs to human protein microarrays.** (**A**) Pfam protein family domains and motifs present in proteins bound to at least 1 RNA (**B**) Gene ontology (GO) terms present in the same population of RNA binding proteins from (**A**). (**C**) Binary heat map representation of the RNA binding capacity to protein microarrays. Human coding and non-coding RNAs used in incubation to the microarrays are displayed in columns. The 137 out of 9400 total proteins bound by sense but not antisense strand RNA samples with ≥ 2.5-fold above background intensity and Z-Score ≥ 3 are displayed in rows. Stau1 and CNBP protein localization indicated with arrows. (**D**) Image and quantification of human protein microarray showing selective binding signal of *TP53* mRNA sense strand to duplicate Stau1 protein spots. The binding signal is shown with respect to adjacent proteins spotted in the same sub-array.

Next, functional characterization of the proteins that significantly bound to RNAs used in this study was performed. Of the 9125 spotted proteins – not including spotted protein controls – only 196 proteins significantly bound to at least 1 of the 20 RNAs. These 196 RNA binding proteins were strongly enriched for protein family domains compiled in the Pfam database that have previously been identified in RNA binding, including the RNA recognition motif, RNA binding domain, zinc finger and K homology domains (Figure 2A). Gene Ontology (GO) terms associated with RNA binding, RNA processing, and RNA splicing among others were strongly enriched (Figure 2B). Taken together, these findings demonstrate the selectivity of this technique to detect RNA-protein interactions.

We selected a subset of these proteins that most frequently bound RNAs (≥75% of all RNAs used) for further domain analysis (Table 1). Interestingly, while 23 out of 28 of these “common RNA binding proteins” contain motifs or domains previously characterized for RNA binding capacity, several proteins such as UNG, KCNAB1, STK40, PAGE1 and NUDT16L1 contain no well-characterized RNA interaction domains despite repeated significant RNA-protein binding events observed in at least 15 out of 20 RNA incubations to the protein array (Table 1). Among these proteins, NUDT16L1 contains no defined domain at all and PAGE1 has no known function except its specific expression in variety tumors. In addition, a comparison of the 28 common RNA binding proteins to those identified by recent PAR-CL and PAR-CLIP-seq studies [19,20] demonstrates that 21/28 and 18/28 of these proteins, respectively, demonstrate RNA binding ability including NUDT16L1.

**Table 1:** Common RNA binding proteins and their conserved domains

**Gene name**	**Protein description (NCBI Protein)**	**Conserved**	**% of all RNAs significantly bound**
		**Domains**	
*CIRBP*	Cold-inducible RNA-binding protein	RRM	90%
*SFRS13A*	FUSIP1 protein	RRM	85%
*CPEB4*	CPEB4 protein	RRM	85%
*PTBP2*	Polypyrimidine tract binding protein 2	RRM	80%
*TIA1*	TIA1 protein	RRM	80%
*TIAL1*	TIAL1 protein	RRM	75%
*RBMS3*	RNA-binding motif, single-stranded-interacting protein 3	RRM	95%
*PTBP1*	Polypyrimidine tract-binding protein 1 isoform c	RRM	95%
*PCBP2*	Poly(rC)-binding protein 2 isoform b	PCBP_like_KH, KH-I	100%
*NOVA1*	RNA-binding protein Nova-1 isoform 1	PCBP_like_KH,KH-I	90%
*PCBP2*	Poly(rC)-binding protein 2 isoform a	PCBP_like_KH, KH-I	90%
*PCBP1*	Poly(rC) binding protein 1	PCBP_like_KH, KH-I	80%
*QKI*	Protein quaking isoform HQK-6	SF1_like-KH	95%
*QKI*	Protein quaking isoform HQK-7B	SF1_like-KH	90%
*QKI*	Protein quaking isoform HQK-5	SF1_like-KH	80%
*KHDRBS2*	KH domain-containing,RNA-binding protein 2	SF1_like-KH	85%
*KHDRBS3*	KHDRBS3 protein	SF1_like-KH	75%
*ZNF385b*	ZNF385B protein	Zf-met	90%
*MBNL1*	Muscleblind-like protein 1 isoform a	Zf-CCCH	75%
*ZC3H10*	Zinc finger CCCH domain-containing protein 10	Zf-CCCH	95%
*TARBP2*	RISC-loading complex subunit TARBP2 isoform b	DSRM	95%
*TARBP2*	RISC-loading complex subunit TARBP2 isoform a	DSRM	100%
*RPA1*	Replication protein A 70 kDa DNA-binding subunit	RPA1N,RPA1_DBD_C	75%
*UNG*	Uracil-DNA glycosylase isoform UNG2	UDG_F1	95%
*KCNAB1*	Voltage-gated potassium channel subunit beta-1 isoform 1	Aldo_ket_red, Tas	95%
*STK40*	Serine/threonine-protein kinase 40	PKc_like	75%
*PAGE1*	G antigen family B member 1	GAGE	75%
*NUDT16L1*	Protein syndesmos isoform 1	None	75%

RRM – RNA recognition motif, PCBP_like_KH – Poly r(C) binding protein like K homology domain, KH – K homology RNA binding domain, KH-I – K homology RNA binding domain type I, SF1_like-KH – Splicing factor K homology RNA binding domain, Zf-CCCH – Zinc-finger of CCCH [C-x8-C-x5-Cx3-H] type, DSRM – Double-stranded RNA binding motif, Aldo_ket_red – Aldo-keto reductase, Tas – predicted oxidoreductase, UDG_F1 – family 1 of Uracil-DNA glycosylase, RPA1N – replication protein A N-terminal OB fold domain, RPA1_DBD_C – replication protein ssDNA binding domain DBD-C, PKc_like – Protein Kinase C family, GAGE – GAGE protein family.

RNA application to human protein microarrays therefore identified a set of human proteins with a broad capacity for binding to multiple RNAs that contain known RNA binding motifs as well as identified a number of new proteins for future study that contain no canonical motifs and no previously known RNA binding capacity. To further narrow the lists of candidate RNA-protein interactions, we selected proteins that significantly bound sense, but not antisense RNAs, although some biologically relevant interactions may take place with both sense and antisense transcripts from a given locus. This analysis resulted in 137 interactions, revealing a substantial range of protein binding between individual mRNAs and non-coding RNAs and identified specific interactions for further study (Figure 2C).

**Figure 3:**
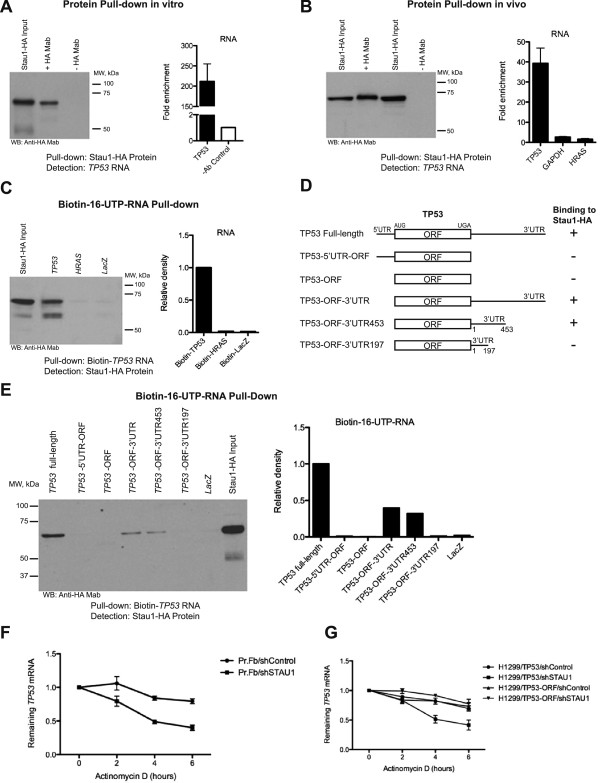
**Confirmation of RNA-protein binding on microarrays with reciprocal pull-down assays for Staufen1 with *TP53 *mRNA.** (**A**) Stau1-HA protein pulls down *TP53* mRNA in vitro after immunoprecipitation with HA Mab; immunoblots to HA-tagged Stau1 verifying Stau1 precipitation are shown on the left panel. (**B**) Stau1-HA protein pulls down *TP53* mRNA in vivo, but not *HRAS* and *GAPDH* control RNAs after immunoprecipitation with HA Mab; immunoblots to HA-tagged Stau1 verifying Stau1 precipitation from cell extracts are shown on the left panel. (**C**) Pull-down of biotin labeled human *TP53* mRNA in vitro, but not *HRAS* or *Lac*Z mRNA precipitates associated Stau1-HA protein; densitometry quantification of the immunoblots shown on the right panel. (**D**) Schematic diagram of *TP53* mRNA constructs. Numbering corresponds to the first nucleotide following the termination codon, defined as 1. Signs (“+” and “–“) represent ability or failure of Stau1-HA to bind *TP53* RNA constructs. (**E**) Pull-down of biotin labeled human *TP53* mRNA deletion constructs in vitro, followed by western blot analysis of the associated Stau1-HA protein; densitometry quantification of the immunoblots shown on the right panel. (**F**) *TP53* RNA decay in Primary Fibroblasts after actinomycin D treatment. (**G**) Comparison of full-length *TP53* and *TP53*-ORF (lacking Staufen 1 interaction domain) RNA decay in TP53 negative H1299 cells after actinomycin D treatment.

To perform biochemical validation of our findings we selected 2 of these newly identified interactions and studied RNA-protein binding using reciprocal pull-down experiments. First, Staufen1 (Stau1) was identified as a protein-binding target of *TP53* mRNA using protein microarrays with signal over background 3.19-fold, Z-score 4.24 (Additional file 3: Table S2). *TP53* mRNA, but not its antisense or any other RNA transcripts, displayed a specific interaction with the duplicate Stau1 protein spots (Figure 2C and 2D). Antibody to an HA epitope-tagged Stau1 protein immunoprecipitated *TP53* mRNA but not control RNAs in both in vitro and in vivo RNA-protein pull-down assays (Figure 3A and 3B). Conversely, using reciprocal RNA pull-down experiments, biotin-labeled *TP53* mRNA – but not biotin-labeled controls – pulled down Stau1 protein (Figure 3C). Therefore, two-way pull-down experiments confirmed the novel *TP53* mRNA-Stau1 protein interaction identified using human protein arrays.

A second interaction validation was undertaken for the RING-type CCHC-zinc finger, nucleic acid binding protein (CNBP), which bound *HRAS* mRNA on the protein microarray with signal over background 3.62-fold, Z-score 3.82 (Figure 2C, Additional file 3: Table S2 and Additional file 4: Figure S2A). The biotin-labeled *HRAS* mRNA, but not biotin-labeled controls, pulled down CNBP protein (Additional file 4: Figure S2B). Moreover, in the complementary pull-down experiments, antibody to HA epitope-tagged CNBP protein immunoprecipitated *HRAS* mRNA but not control RNAs both in vitro (Additional file 4: Figure S2C), as well as in vivo (Additional file 4: Figure S2D). These two-way studies of two independent, novel targets support the validity of RNA-protein interactions detected by RNA incubation to the human protein microarray and further verifies the capability of this method.

To further address significance of *TP53* association with Stau1 protein, several deletion mutants spanning the UTRs of *TP53* RNA were generated and used for Stau1-HA protein pull-down (Figure 3D and 3E). Although we could not identify any known Stau1 binding sequence motifs [21] within the *TP53* UTR, pull-down experiments indicated that Stau1 protein preferentially binds within a 256 bp sequence of the *TP53* 3^′^UTR. Moreover, repeated incubation of the TP53 open reading frame to the protein array demonstrated absence of binding signal to Stau1 duplicate spots (Additional file 4: Figure S2E), while the full-length TP53 RNA showed binding (Figure 2D), confirming the finding that Stau1 protein binding takes place within the *TP53* 3^′^UTR.

Stau1 protein is known to be involved in several cellular functions including RNA decay (in combination with UPF1) [22], RNA transport [23], RNA translation [24] and modulation of stress response [25] via shuffling between polysomes and stress granules. To explore consequences of Stau1 binding to *TP53* RNA we studied *TP53* RNA stability in *STAU1* and *UPF1* deficient cells. Unexpectedly, we found that although *UPF1* depletion did not affect overall *TP53* RNA levels (Additional file 4: Figure S2F), *STAU1* knock down caused reduction in *TP53* RNA half-life in actinomycin D treated primary fibroblast cells, under conditions known to halt synthesis of newly formed RNA molecules (Figure 3F). Moreover, after introduction of the full-length *TP53* and *TP53*-ORF (lacking 3^′^UTR and Stau1 protein binding site) in TP53 negative H1299 cells, STAU1 knock down caused reduction in the full-length *TP53* RNA half-life but did not affect *TP53*-ORF RNA after actinomycin D treatment (Figure 3G). These initial findings indicate that Stau1 protein binding to *TP53* RNA may play a role in preserving *TP53* RNA levels in the setting of a transcriptional blockade.

The diversity of naturally occurring RNA-protein interactions is only beginning to be appreciated and RNA hybridization to human protein microarrays may be a useful complement to current platforms in a number of ways. For example, this approach can be used to gain mechanistic insight into newly identified long non-coding RNAs by understanding the proteins to which they bind. The ability to test the binding capability of any coding or non-coding RNA to thousands of proteins simultaneously will significantly improve the pace of mechanistic analyses of RNAs. In this regard, it may provide a complement to biotin-labeled RNA pull-down/mass spectrometry-based approaches, which have proven successful in identifying RNA binding proteins [8] but require substantial amounts of cell-derived starting material and are laborious and time-consuming. RNA hybridization to human protein microarrays, in contrast, requires minimal amounts of RNA and can be completed in less than one day. However, the use of entirely recombinant components for this method precludes the detection of RNA-protein interactions that 1) require intact protein complexes, 2) require post-translational modifications of protein, and 3) take place with proteins not spotted on the array.

RNA hybridization to human protein microarrays may also help to understand proteins that bind and regulate the stability, localization, or translational control of protein coding transcripts. In this regard, Stau1, a known RNA-binding protein implicated in both RNA stability and localization [22-24], was shown in this work to regulate *TP53* in an as yet previously uncharacterized way. Similarly, the binding of CNBP to *HRAS* RNA may also modulate Ras function. Moreover, it is intriguing to note that human genetic disorders arising from mutations in both *CNBP* (Myotonic Dystrophy [26]*)* and *HRAS* (Costello Syndrome [27]), while displaying distinctive features, both display profound abnormalities in muscle tissue. Similarly, we observed the Prader-Willi Syndrome associated long non-coding RNA, PWRN1, to bind the SPATA17 protein, which has been linked to apoptosis of spermatogenic cells [28]. As hypogonadism is a known symptom of Prader Willi Syndrome, this observation may provide a functional link between this non-coding RNA and its associated human disease. Incubation of specific human RNAs of interest to human protein microarrays may therefore help characterize mechanisms of RNA function and may also stimulate efforts to identify potential uncharacterized links in the pathogenesis of human disease.

## Conclusions

Here we describe a refined methodology for rapid and large-scale identification of novel human RNA-protein interactions. RNA hybridization to human protein microarrays described here offers several attractive features. First, the reagents required are readily available, consisting of minimal amounts of in vitro transcribed RNA and standardized, commercially available protein microarrays. Second, RNA hybridization to human protein microarrays does not require large-scale cell culture for protein isolation and mass spectrometry, and hence it is far less laborious than current RNA chromatography techniques. Third, this technique is rapid, taking less than a day to complete. Biochemical verification of newly identified RNA-protein interactions using this technique via reciprocal and independent pull-down experiments performed here moreover suggests that there are many undiscovered human-RNA protein interactions and that this approach may be helpful in identifying them.

## Methods

### Plasmid vectors and expression constructs

Plasmid vectors containing full-length transcribed sequences for coding and non-coding RNAs were obtained from Open Biosystems (Thermo Scientific) and are described in detail in Additional file 2: Table S1. All plasmids except pDNRLIB contain T7, T3 or SP6 promoters for sequencing and in vitro RNA production. pDNRLIB-DLEU1 plasmid contains the T7 promoter for sense RNA transcription. To produce antisense DLEU1 RNA, a 945 bp DLEU1 sequence was recloned from pDNRLIB into pSPORT1 vector using EcoRI and XhoI sites and pSPORT1-DLEU1 was used for antisense RNA production via SP6 promoter. For in vivo RNA production we constructed a small eukaryotic expression vector pSPARTA containing the human PGK promoter controlling expression of the transcribed sequence and puromycin resistance gene for selection in both prokaryotes and eukaryotes, which is under control of the synthetic bacterial EM7 and viral CMV promoter (Additional file 1: Figure S1). The *TP53* mRNA sequence [GenBank:NM_000546] was recloned from the original pSPORT1-TP53 vector as a SalI-ClaI fragment of 2560 bp into SalI-EcoRV of pSPARTA. The TP53 deletion mutants containing 5^′^UTR and 197bp of 3^′^UTR were constructed by flanking TP53 fragments with either HindIII-XhoI or EcoRI-XhoI and cloning into pcDNA3 expression vector to generate pcDNA3-TP53-ORF-5^′^UTR and pcDNA3-TP53-ORF-3^′^UTR197. The constructs containing TP53-ORF, TP53-ORF-3^′^UTR (full-length) and TP53-ORF-3^′^UTR453 fragments were generated by direct amplification and TA cloning into pcDNA3.1TOPO expression vector. Numbering for 3^′^UTR deletion constructs corresponds to the first nucleotide following the termination codon, defined as 1. The full-length TP53 expression construct used for transfection of H1299 cells was constructed by recloning HindIII-NotI fragment from pSTARTA-TP53 into pcDNA3 in order to take advantage of the neomycin selection cassette in H1299 cells.

Human V-Ha-Ras homolog [GenBank:BC006499] was recloned from pOTB7 vector as 1146 bp EcoRI-XhoI fragment into EcoRV-XhoI of pSPARTA. Both plasmids pSPARTA-TP53 and pSPARTA-HRAS were used in pull-down experiments in vivo as described below. In vivo expression constructs for Stau1 and CNBP were generated using pcDNA3.1Hygro plasmid (Invitrogen). The ORFs of Stau1 and CNBP were flanked with 3x hemagglutinin (HA) tag at 3^′^ end for Stau1 and 5^′^ end for CNBP using PCR techniques and the following primers:

STAUF, 5^′^TTTTAAGCTTACCATGTCTCAAGTTCAAGTGCAAGTT.

STAUHAR, 5^′^TTTTCTCGAGTCAGGCGTAATCGGGCACGTCGTA

GGGATAGCTTCCTGCATAATCAGGGACGTCATAGG

GATAGCCAGCATAGTCAGGCACATCGTATGGGTAG

CACCTCCCACACACAGACA.

CNBPHAF, 5^′^TTTTAAGCTTACCATGTACCCATACGA

TGTGCCTGACTATGCTGGCTATCCCTATGACGTCC

CTGATTATGCAGGAAGCTATCCCTACGACGTGCCC

GATTACGCCAGCAGCAATGAGTGCTTCAAG.

CNBPR, 5^′^TTTTCTCGAGTTAGGCTGTAGCCTCAATTGTG.

The epitope tagged fragments were cloned in HindIII-XhoI sites of pcDNA3.1Hygro and the final constructs pcDNA3.1Hygro-STAU1-HA and pcDNA3.1Hygro-HA-CNBP were used for RNA pull-down studies.

### *In vitro* RNA production and labeling

For the 10 RNA expression sequences used in this study, RNAs for coding (*TP53*, *MYC*, *HRAS*, *BCL2*) and non-coding (*OCC1*, *IGF2RNC*, *PWRN1*, *DLEU1*, *lncRBM26, SOX2OT*) genes were in vitro transcribed in both sense and antisense directions using T7, T3 or SP6 promoters (Additional file 2: Table S1). First, plasmid DNA was digested with enzymes immediately flanking the transcribed sequence and 4 μg of linear DNA was used for in vitro transcription in 50 μl total volume consisting of: 1x Transcription buffer (Promega), 10 mM DTT (Promega), 1 mM NTP (Invitrogen), 40 units RNAseOUT (Invitrogen) and 60 units RNA polymerases (T7, T3 or SP6). The reaction was carried out at 37°C for 4 hours after which DNA was digested by addition of 2 units DNAse I at 37°C for 15 minutes. Next, RNA was phenol-chloroform extracted and after ethanol precipitation measured using NanoDrop 1000 spectrophotometer (Thermo Scientific) and visualized using denaturing agarose gel-electrophoreses (Additional file 1: Figure S1C).

RNA labeling for microarray incubations was performed using *Label* IT μArray Cy5 labeling kit (Mirus). We first carefully optimized the labeling procedure in order to achieve between 1 to 3 Cy5 dyes covalently attached to RNAs used. This was accomplished via the following modifications from the original manufacturers protocol: the total reaction volume was kept at 25 μl, the ratio of RNA:*Label* IT Cy5 reagent at 10:1 (w:v) and reaction time not more then 30 minutes at 37°C. Briefly, 5 μg RNA in water was mixed with 5 μl *Label* IT Cy5, diluted 1 to 10 in water to obtain a final volume of 25 μl and incubated 30 minutes at 37°C. The reaction was stopped by addition 2.5 μl of 10x STOP buffer (Mirus). The volume was increased to 100 μl with water, supplemented with glycogen (Invitrogen) to final concentration of 0.2 μg/μl, mixed and RNA ethanol precipitated in the presence of 0.5 M NaCl at −20°C for at least 1 hour. The labeled RNA was extensively washed in 70% ethanol, dried and resuspended in 16 μl water. RNA labeling density was evaluated using NanoDrop 1000 spectrophotometer (Thermo Scientific) and visualized using denaturing agarose gel-electrophoreses.

The efficacy of Cy5 dye incorporation was calculated as dye density (pmol Dye: μg RNA) and RNA Base:Dye ratio. To calculate the dye density following formula was used:

pmol Dye : μg RNA = A_dye_/e_dye_(pmol)/M_RNA_, (μg), where

A_dye_ – Cy5 absorbance at I_max_ (excitation wavelength) 649 nm,

e_dye_ – Cy5 dye extinction coefficient 250 000 M^-1^ cm^-1^,

M_RNA_ – RNA amount (μg).

Base:Dye ration was calculated using following formula:

(A_base_x e_dye_)/(A_dye_x e_base_), where

A_base_ = A_260_ – (A_dye_ x C.F._260_) – RNA base absorbance,

A_260_ – absorbance of the nucleic acid, C.F._260_ – correction factor for Cy5=0.05,

e_base_ – RNA extinction coefficient 8250 M^-1^ cm^-1^

The RNA labeling density and Base/Dye labeling ratio for each 20 sense and antisense RNAs used in this work is presented in Additional file 2: Table S1, with an RNA labeling efficacy required of 1 Cy5 dye per 700 – 1200 bp RNA.

RNA labeling with Biotin-16-UTP was performed during in vitro transcription. First, plasmid DNA containing transcribed sequence was digested with enzymes immediately flanking the insert and 4 μg of linear DNA was used for RNA biotinilation reaction in 50 μl total volume consist of: 1x Transcription buffer (Promega), 1 mM ATP, 1mM CTP, 1mM GTP, 0.95 mM UTP, 0.05 mM Biotin-16-UTP (Roche), 10 mM DTT (Promega), 40 units RNAseOUT (Invitrogen) and 60 units RNA polymerases (T7, T3 or SP6). The reaction was carried out at 37°C for 4 hours after which DNA was digested by addition of 2 units DNAse I at 37°C for 15 minutes. Next, RNA was phenol chloroform extracted, and after ethanol precipitation, characterized using NanoDrop 1000 spectrophotometer (Thermo Scientific) and visualized using denaturing agarose gel-electrophoreses (Additional file 1: Figure S1D).

### ProtoArray processing and analysis

For RNA incubation, ProtoArray Human Protein Microarray v5.0 (Invitrogen, cat# PAH052520) was used. Prior to incubation, each microarray was equilibrated first to 4°C overnight and then to 25°C for at least for 15 minutes. The microarray slide was assembled in a Gentel SIM*plex* 16 Multi-Array System device (Gentel biosciences, cat# 4–1007) with custom modifications including a modified bottom gasket and top spacers (Additional file 1: Figure S1A). The bottom gasket was cut out from silicone slab as a rectangular seal with the following dimensions in mm: outer 65L × 25W × 4H, inner 59L × 20W × 4H. Top spacers were cut out from a polycarbonate piece as a rectangular shape with the following dimensions in mm: Upper spacer 5L × 22W × 1H, lower spacer 12L × 22W × 1H. First, the microarray slide was placed inside a Gentel SIM*plex* 16 Multi-Array device bottom holder piece, next the silicone gasket was carefully placed on the top of the slide and the device top piece held in place with tightening screws. The slide surface was blocked in 0.7 mL blocking buffer BL: 40 mM Tris–HCl (pH 8.0), 1% BSA (w/v) (globulin free, Sigma, cat# A7638), 100 μg/ml Yeast tRNA, 20 μg/mL heparin and 1 mM DTT for 1 hour at room T°C with gentle agitation. After completion of the blocking step, 10 pmol Cy5 labeled RNA was added to 0.7 mL binding buffer BB: 40 mM Tris–HCl (pH 8.0), 150 mM sodium chloride, 0.5 mM magnesium acetate, 10 μg/ml Yeast tRNA, 10 μg/mL heparin, 1 mM DTT, 0.01% Igepal CA-630, 5% glycerol, 0.2 units/μl RNAseOUT. The blocking buffer was replaced with binding buffer containing labeled RNA and microarray slides incubated in the dark at 25°C for 1 hour with gentle agitation. Following RNA incubation binding buffer was removed and washing steps were implemented using 3 times exchange of 0.75 mL WB buffer: 40 mM Tris–HCl (pH 8.0), 150 mM sodium chloride, 0.5 mM magnesium acetate, 10 μg/ml Yeast tRNA, 10 μg/mL heparin, 1 mM DTT, 0.01% Igipal 40, 5% Glycerol, 0.2 units/μl RNAseOUT for at least 5 minutes each. Finally, the microarray slide was washed in 0.75 mL washing buffer WBF: 40 mM Tris–HCl (pH 8.0), 150 mM sodium chloride, 0.5 mM Magnesium acetate, 10 μg/ml Yeast tRNA, 10 μg/mL heparin, 1 mM DTT, at least 3 times for 5 minutes each. After the last wash, the WBF buffer was removed partially in order to prevent the slide from drying prematurely and slide holder device disassembled immediately. The probed microarray slide was placed in ArrayIt microarray high-speed centrifuge and the residual WBF buffer removed via a 30 seconds of centrifugation. The dry slide was scanned at 635 nm (Cy5) using a GenePix 4000B Microarray scanner (Molecular Devices) immediately after or at least within 2 hours of the completion of the incubation. All raw and processed data is publicly available at the Gene Expression Omnibus under accession GSE34794 ( http://www.ncbi.nlm.nih.gov/geo/query/acc.cgi?token=ttehdwscqcgckrg&acc=GSE34794). For the visualization process, the array images from antisense RNA incubations were pseudocolored green and overlaid with the sense RNA incubation. The intensity of the 635 nm wavelength signal at each spotted protein location was determined with GenePix Pro 6.1 software (Molecular Devices). To quantify RNA-protein interactions, the intensity of 635 nm signal (F635) was divided by the local background intensity (B635) at each of the duplicate spots for a given protein. Data was filtered based on signal above the background for each of the duplicate feature to be greater than 2.5 fold and Z-Score ≥ 3 from the global mean signal from all of the spotted proteins. In order to select sense-specific protein interactions, the signal generated from antisense RNA incubations were calculated based on 2.5 fold above the background and Z-Score ≥ 3. Significant hits from the antisense were subtracted from the significant hits in the sense RNA. Hierarchical clustering analysis was performed by Cluster 3.0 (Eisen Lab) and visualized with TreeView. Gene Ontology analysis and PFAM domain analysis of RNA binding proteins was performed with DAVID [29] using as a background a universe of gene list of all proteins spotted on the microarray and Benjamini-Hochberg correction of the p value. The p value of the Venn diagram illustrating overlap of two independent microarray incubations was calculated using Fisher’s exact test.

### RNA-protein complex pull-downs

RNA-protein complex pull-downs were performed in two complementary directions: first, by immunoprecipitation of target protein with following associated RNA detection using qPCR technique. Second, by pulling-down biotin labeled RNA and detecting RNA associated protein via western blot analyses.

#### *In vitro* protein IP with subsequent RNA detection

For in vitro protein RNA complex immunoprecipitation both Stau1-HA and HA-CNBP proteins were in vitro translated using rabbit reticulocyte lysate system (Promega) in accordance with manufacturer’s recommendations. The full-length human *TP53* and *HRAS* mRNA transcripts were in vitro transcribed as described in above. Prior to RNA-protein complex formation, 20 μl Protein G Dynabeads (Invitrogen) were saturated in 500 μl buffer IPB containing 40 mM Tris–HCl (pH 8.0), 150 mM sodium chloride, 0.5 mM Magnesium acetate, 20 μg/mL heparin, 1 mM DTT, 0.01% Igepal CA-630, 5% Glycerol, supplemented with 0.5% BSA, 100 μg/ml Yeast tRNA and protease inhibitors complete mini (Roch) and mixed with 2.5 μg anti-HA antibody (HA.11, clone 16B12, Covance) for 1 hour with continuous agitation at 25°C. 12.5 μl of in vitro translated protein was mixed with 250 ng RNA in 500 μl buffer IPB supplemented with 0.2 units/μl RNAseOUT and incubated 1 hour at 25°C with gentle agitation. After one wash of HA-bound protein G Dynabeads in IPB buffer protein-RNA complex was added to the beads and incubated for 1 hour at room T°C. As a control, pre-blocked protein G Dynabeads lacking HA antibody was added to the same amount of protein-RNA complex and processed identically to the sample tube. After completion of the protein capture on Dynabeads, five separate washes for ten minutes duration each were implemented using IPB buffer. Magnetic beads were resuspended in 100 μl IPB and 10% removed for protein analysis via western. The RNA from the residual mixture containing protein-RNA complex was extracted with phenol-chloroform then ethanol precipitated for resuspension in 11.5 μl water in order to use for cDNA synthesis with iScript cDNA Synthesis kit (Bio-Rad). After completion of the reverse transcription, DNA samples were subjected to qPCR using Maxima SYBR Green qPCR master mix (2x, Fermentas) and gene specific primers sets:

TP53_F: 5^′^CCAGCCAAAGAAGAAACCAC

TP53_R: 5^′^TGAGTTCCAAGGCCTCATTC

HRAS_F: 5^′^AGCAGATCAAACGGGTGAAG

HRAS_R: 5^′^AGCCAGGTCACACTTGTTCC

The qPCR was performed on Stratagene Mx3000P QPCR system (Agilent Technologies) and results were analyzed with MxPro QPCR software v. 4.1.

#### *In vivo* protein IP with subsequent RNA detection

For in vivo protein-RNA complex pull-down experiments 293T cells were transfected with expression vectors pcDNA3.1Hygro-STAU1-HA, pSPARTA-TP53, and pcDNA3.1Hygro-HA-CNBP pSPARTA-HRAS in 10 cm plates using FuGENE 6 transfection reagent (Roche) in accordance with the manufacturer’s instructions. Cells were collected 48 hours post-transfection in 2 volumes (v/v cell pallet) of Buffer A: 10 mM Tris–HCl (pH 7.5), 0.1 mM EDTA, 1 mM DTT, 1 mM PMSF and protease inhibitors complete mini (Roch) and incubated 15 minutes on 4°C. While cells were incubated on ice Protein G Dynabeads were blocked and bound to HA mab as described above using IPB buffer. After 15 minutes Igepal CA-630 was added to cells to a final concentration of 0.01% and incubated additional 5 minutes at 4°C. Next, cells were subjected to two freeze-thaw cycles by incubating on isopropanol/dry ice bath for 30 seconds and immediately thawing at 37°C. After completion of the lyses, 1.5 μl of RNAseOUT (Invitrogen) was added for every 100 μl lysate and subjected to centrifugation at 1,000g for 5 minutes at 4°C. The cytosolic fraction was removed and kept at 4°C, while to remaining pellet Buffer B was added equal to the volume of original Buffer A supplemented with 2.5 units of DNAse I for every 100 μl of original cell pellet. Buffer B is composed of: 50 mM Tris–HCl (pH 7.5), 300 mM sodium chloride, 1 mM Magnesium acetate, 1 mM DTT, 10% Glycerol (v/v), 1 mM PMSF and protease inhibitors complete mini (Roch). The lysate was passed through 27 gauge needle, spun at 10,000g for 15 minutes at 4°C and, after adding Igepal CA-630 to a final concentration of 0.01%, combined with cytosolic fraction. Next, the combined lysate was split in two parts and treated with 20 μl Protein G Dynabeads with or without HA Mab for 1 hour at room T°C. The magnetic beads were subjected to at least 5 washing cycles using 500 μl IPB buffer and, after collection in 100 μl buffer, 1/10 of the volume was saved for protein analysis via western and from the rest of the pull-down RNA extracted using TRIzol reagent (Invitrogen) in accordance with the manufacturer’s instructions. The RNA pallet was resuspended in 11.5 μl in order to use for cDNA synthesis with iScript cDNA Synthesis kit (Bio-Rad). After completion of the reverse transcription, DNA samples were subjected to qPCR using Maxima SYBR Green qPCR master mix (2x, Fermentas) and gene specific primers sets indicated above together with the control primers:

GAPDH_F: 5^′^ GAAGAGAGAGACCCTCACTGCTG

GAPDH_R: 5^′^ACTGTGAGGAGGGGAGATTCAGT

The qPCR was performed on Stratagene Mx3000P QPCR system (Agilent Technologies) and results were analyzed with MxPro QPCR software v. 4.1.

#### *In vitro* RNA pull-down with subsequent protein detection

For in vitro RNA pull-down *TP53* full-length or deletion mutants TP53-5^′^UTR-ORF, TP53-ORF, TP53-ORF-3^′^UTR, TP53-ORF-3^′^UTR453, TP53-ORF-3^′^UTR197, *HRAS* and *Lac*Z mRNAs were labeled with biotin-16-UTP as described above and Additional file 1: Figure S1D. The control *Lac*Z RNA was prepared as 1200 bp fragment of the full-length *Lac*Z via in vitro transcription of the EcoRV digested pcDNA3.1HygroLacZ (Invitrogen) with T7 polymerase in order to match the average length of the sample RNAs used in this work. The 5 μl in vitro translated Stau1-HA or HA-CNBP was incubated with 1 μg biotin-16-UTP labeled *TP53*, *HRAS*, or *Lac*Z in IPB buffer for 30 minutes at 25°C. During this reaction, 5 μl of MyOne Streptavidin T1 Dynabeads (Invitrogen) were exchanged to IPB buffer using magnetic stand and added to protein RNA complex. The mixture was incubated an additional 30 minutes and subjected to five wash cycles of 5 minutes each using 500 μl IPB buffer. After the last wash, magnetic beads were resuspended in 12 μl protein loading buffer, RNA bound protein separated by SDS-PAGE and detected with anti-HA Mab by western blot analysis.

### RNA interference

siRNA oligonucleotide duplexes used in this work were synthesized by Dharmacon (Thermo Scientific). 1x10^6^ Pimary human Fibroblasts were electroporated with 1 nmol siRNA nucleotides using Amaxa Human Dermal Fibroblast nucleofection kit (Lonza) following manufacturer’s instruction. The siRNA oligonucleotides were used in this work:

siControl: 5^′^ GUAGAUUCAUAUUGUAAGGUU

siUPF1 A: 5^′^ CAGCGGAUCGUGUGAAGAA

siUPF1 C: 5^′^ GCAGCCACAUUGUAAAUCA

The vectors for pGIPZ shRNA targeting Stau1 were designed and purchases through Open Biosystems (Thermo Scientific) catalog number RHS4531-NM_017452. The efficiency of knockdown was evaluated using following oligonucleotides:

STAU_F: 5^′^ ATGGTATCGGCAAGGATGTG

STAU_R:5^′^ AGACATTGGTCCGTTTCCTG

UPF1_F: 5^′^ ATATGCCTGCGGTACAAAGG

UPF1_R: 5^′^ AGCTCAATGGCGATCTCATC

### Tissue culture

All of the experimental procedures were carried out in accordance with the local ethics commission. Primary human dermal fibroblasts were isolated from neonatal dermis and cultured at early passage in DMEM supplemented with 10% FBS. H1299 non-small cell lung carcinoma cell line deficient for TP53 was cultured in RMPI1640 supplemented with 10% FBS. H1299 cells were transfected with pcDNA3-TP53 full-length or pcDNA3.1TOPO-TP53-ORF constructs and selected for 3 to 5 days in 500 μg/mL neomycin. Next, cells were transduced with pGIPZ-STAU1 virus and 36 hours later selected with 1 μg/mL puromycin for 48 hours. For RNA stability analysis, cells were plated 24 [hour]s before at 40-50% confluence in 6 well plate and next day treated with 5 μg/mL actinomycin D (Sigma) for the indicated times. Total RNA was prepared and gene expression analyses as described above.

## Abbreviations

RNA: Ribonucleic acid; RIP-Chip: RNA-binding protein immunoprecipitation-microarray profiling; RIP-Seq: RNA-binding protein immunoprecipitation-sequencing; RRM: RNA recognition motif; RNP: Ribonucleic acid-protein; UTR: Untranslated region; PGK: Phosphoglycerate kinase; EM7: Prokaryotic promoter; CMV: Cytomegalovirus; ORF: Open reading frame; PCR: Polymerase chain reaction; UTP: Uridine-5^′^-Triphosphate; ATP: Adenosine-5^′^-Triphosphate; CTP: Cytidine-5^′^-Triphosphate; GTP: Guanosine -5^′^-Triphosphate; DTT: Dithiothreitol; Tris: 2-Amino-2-hydroxymethyl-propane-1,3-diol; tRNA: Transfer RNA; EDTA: Ethylenediaminetetraacetic acid; PMSF: Phenylmethanesulfonylfluoride; SDS-PAGE: Sodium dodecyl sulfate polyacrylamide gel electrophoresis; PFAM: Database of protein families; GO: Gene ontology; DAVID: The Database for annotation, visualization and integrated discovery.

## Competing interests

The authors declare no competing financial interests.

## Authors’ contribution

ZS and DJ carried out experiments including RNA labeling, protein array probing and functional studies, DW participated in data analysis and statistical calculations, MK participated in the design of the study and RNA interference experiments. PK directed the research, and with JR and HC, conceived and participated in its design, coordination and helped to write the manuscript. All authors read and approved the final manuscript.

## Supplementary Material

Additional file 1 **Figure S1.** Protein microarray incubation device and RNAs used for this work. (**A**) Modified Gentel SIMplex 16 device with microarray slide and assembly components. Schematic diagram of the custom-made silicone gasket and spacer with main dimensions indicated. (**B**) Expression vector pSPARTA. hPGK - human phosphoglycerate kinase promoter, SV40 polyA - simian virus 40 polyadenylation signal, bGlob polyA - beta-globin polyadenylation signal, Puromycin - resistance gene, EM7 - bacterial promoter, CMV Enh/Prom - cytomegalovirus enhancer promoter, Ori - origin of replication. Unique site depicted in black. Polylinker sites are in red. (**C**) Denaturing agarose gel electrophoresis of sense and antisense RNAs used in this work. M - RiboRuler RNA ladder (bp): 6000, 4000, 3000, 2000, 1500, 1000, 500, 200. (**D**) Denaturing agarose gel electrophoresis of biotin-16-UTP labeled RNAs *TP53*, *HRAS*, *Lac*Z (1.2kb fragment of *Lac*Z, experimental procedures). M - RiboRuler RNA ladder (bp): 6000, 4000, 3000, 2000, 1500, 1000, 500, 200. Click here for file

Additional file 2 **Table S1.** Expression constructs and promoters used for the strand-specific RNA sequence production. The efficiency of RNA labeling with Cy5 dye was calculated as described in Experimental Procedures. Click here for file

Additional file 3 **Table S2.** Significant RNA binding proteins for all sense and antisense RNAs used in this work. Z-Score ≥ 3 and signal intensity above background ≥ 2.5 was used to filter RNA-protein binding events as described in the text. Click here for file

Additional file 4 **Figure S2.** Confirmation of RNA-protein binding on microarrays. The reciprocal pull-down assays for CNBP with *HRAS* mRNA. (**A**) Quantitation images of human microarray showing selective binding signal of *HRAS* mRNA sense strand to duplicate CNBP protein spots. The incubation signal shown with respect to adjacent protein controls in the same sub-array. (**B**) Pull-down of biotin labeled *HRAS* mRNA in vitro, but not *TP53* or *Lac*Z precipitates associated HA-CNBP protein; densitometry quantification of the immunoblots shown (right). (**C**) HA-CNBP protein pulls down *HRAS* mRNA in vitro after immunoprecipitation with HA Mab; immunoblots to HA-tagged CNBP verifying CNBP precipitation are shown on the left panel. (**D**) HA-CNBP protein pulls down *HRAS* mRNA in vivo, but not control *TP53* and *GAPDH* mRNAs after immunoprecipitation with HA Mab; immunoblots to HA-tagged CNBP verifying CNBP precipitation from cell extracts are shown (left). (**E**) Incubation of *TP53*-ORF mRNA sense strand lacking 5′ and 3′ UTR regions to human protein microarrays. Panel at left shows the entire microarray spotted with ~9400 recombinant human proteins; the middle panels represent an enlargement of the sub-array containing Stau1 and WIT1 proteins. Note absence of the Stau1-*TP53* mRNA association signal in comparison to Figure 2D when full-length *TP53* mRNA was probed. [all proteins spotted in duplicate; Stau1 and WIT1 spots boxed in white; sub-array positive controls boxed in red]. The quantification of the incubation results shown on the right. (**F**) Contrary to STAU1 depletion, UPF1 KD has no affect on *TP53* RNA decay in Primary Fibroblasts. Click here for file

## References

[B1] Wilusz JE Sunwoo H Spector DL Long noncoding RNAs: functional surprises from the RNA world Genes Dev 2009 23 13 1494 1504 10.1101/gad.1800909 19571179 PMC3152381

[B2] Mattick JS RNA regulation: a new genetics? Nat Rev Genet 2004 5 4 316 323 10.1038/nrg1321 15131654

[B3] Ponting CP Oliver PL Reik W Evolution and functions of long noncoding RNAs Cell 2009 136 4 629 641 10.1016/j.cell.2009.02.006 19239885

[B4] Bartel DP MicroRNAs: target recognition and regulatory functions Cell 2009 136 2 215 233 10.1016/j.cell.2009.01.002 19167326 PMC3794896

[B5] Pesole G Mignone F Gissi C Grillo G Licciulli F Liuni S Structural and functional features of eukaryotic mRNA untranslated regions Gene 2001 276 1–2 73 81 11591473 10.1016/s0378-1119(01)00674-6

[B6] Mignone F Gissi C Liuni S Pesole G Untranslated regions of mRNAs Genome Biol 2002 3 3 REVIEWS0004 11897027 10.1186/gb-2002-3-3-reviews0004 PMC139023

[B7] Guttman M Amit I Garber M French C Lin MF Feldser D Huarte M Zuk O Carey BW Cassady JP Chromatin signature reveals over a thousand highly conserved large non-coding RNAs in mammals Nature 2009 458 7235 223 227 10.1038/nature07672 19182780 PMC2754849

[B8] Khalil AM Guttman M Huarte M Garber M Raj A Rivea Morales D Thomas K Presser A Bernstein BE van Oudenaarden A Many human large intergenic noncoding RNAs associate with chromatin-modifying complexes and affect gene expression Proc Natl Acad Sci USA 2009 106 28 11667 11672 10.1073/pnas.0904715106 19571010 PMC2704857

[B9] Pauli A Rinn JL Schier AF Non-coding RNAs as regulators of embryogenesis Nat Rev Genet 2011 12 2 136 149 10.1038/nrg2904 21245830 PMC4081495

[B10] Rinn JL Kertesz M Wang JK Squazzo SL Xu X Brugmann SA Goodnough LH Helms JA Farnham PJ Segal E Functional demarcation of active and silent chromatin domains in human HOX loci by noncoding RNAs Cell 2007 129 7 1311 1323 10.1016/j.cell.2007.05.022 17604720 PMC2084369

[B11] Rastan S X chromosome inactivation and the Xist gene Curr Opin Genet Dev 1994 4 2 292 297 10.1016/S0959-437X(05)80056-5 8032207

[B12] Yotova IY Vlatkovic IM Pauler FM Warczok KE Ambros PF Oshimura M Theussl HC Gessler M Wagner EF Barlow DP Identification of the human homolog of the imprinted mouse Air non-coding RNA Genomics 2008 92 6 464 473 10.1016/j.ygeno.2008.08.004 18789384 PMC2846268

[B13] Martianov I Ramadass A Serra Barros A Chow N Akoulitchev A Repression of the human dihydrofolate reductase gene by a non-coding interfering transcript Nature 2007 445 7128 666 670 10.1038/nature05519 17237763

[B14] Townley-Tilson WH Pendergrass SA Marzluff WF Whitfield ML Genome-wide analysis of mRNAs bound to the histone stem-loop binding protein RNA 2006 12 10 1853 1867 10.1261/rna.76006 16931877 PMC1581977

[B15] Hafner M Landthaler M Burger L Khorshid M Hausser J Berninger P Rothballer A Ascano M Jr Jungkamp AC Munschauer M Transcriptome-wide identification of RNA-binding protein and microRNA target sites by PAR-CLIP Cell 2010 141 1 129 141 10.1016/j.cell.2010.03.009 20371350 PMC2861495

[B16] Slobodin B Gerst JE A novel mRNA affinity purification technique for the identification of interacting proteins and transcripts in ribonucleoprotein complexes RNA 2010 16 11 2277 2290 10.1261/rna.2091710 20876833 PMC2957065

[B17] Tsvetanova NG Klass DM Salzman J Brown PO Proteome-wide search reveals unexpected RNA-binding proteins in Saccharomyces cerevisiae PLoS One 2010 5 9 e12671 10.1371/journal.pone.0012671 20844764 PMC2937035

[B18] Scherrer T Mittal N Janga SC Gerber AP A screen for RNA-binding proteins in yeast indicates dual functions for many enzymes PLoS One 2010 5 11 e15499 10.1371/journal.pone.0015499 21124907 PMC2988813

[B19] Baltz AG Munschauer M Schwanhausser B Vasile A Murakawa Y Schueler M Youngs N Penfold-Brown D Drew K Milek M The mRNA-bound proteome and its global occupancy profile on protein-coding transcripts Mol Cell 2012 46 5 674 690 10.1016/j.molcel.2012.05.021 22681889

[B20] Castello A Fischer B Eichelbaum K Horos R Beckmann BM Strein C Davey NE Humphreys DT Preiss T Steinmetz LM Insights into RNA biology from an atlas of mammalian mRNA-binding proteins Cell 2012 149 6 1393 1406 10.1016/j.cell.2012.04.031 22658674

[B21] Kim YK Furic L Parisien M Major F DesGroseillers L Maquat LE Staufen1 regulates diverse classes of mammalian transcripts EMBO J 2007 26 11 2670 2681 10.1038/sj.emboj.7601712 17510634 PMC1888674

[B22] Kim YK Furic L Desgroseillers L Maquat LE Mammalian Staufen1 recruits Upf1 to specific mRNA 3′UTRs so as to elicit mRNA decay Cell 2005 120 2 195 208 10.1016/j.cell.2004.11.050 15680326

[B23] Kiebler MA Hemraj I Verkade P Kohrmann M Fortes P Marion RM Ortin J Dotti CG The mammalian staufen protein localizes to the somatodendritic domain of cultured hippocampal neurons: implications for its involvement in mRNA transport J Neurosci: the official journal of the Society for Neuroscience 1999 19 1 288 297 10.1523/JNEUROSCI.19-01-00288.1999 PMC6782358 9870958

[B24] Dugre-Brisson S Elvira G Boulay K Chatel-Chaix L Mouland AJ DesGroseillers L Interaction of Staufen1 with the 5′ end of mRNA facilitates translation of these RNAs Nucleic Acids Res 2005 33 15 4797 4812 10.1093/nar/gki794 16126845 PMC1193567

[B25] Thomas MG Martinez Tosar LJ Loschi M Pasquini JM Correale J Kindler S Boccaccio GL Staufen recruitment into stress granules does not affect early mRNA transport in oligodendrocytes Mol Biol Cell 2005 16 1 405 420 15525674 10.1091/mbc.E04-06-0516 PMC539183

[B26] Chen W Wang Y Abe Y Cheney L Udd B Li YP Haploinsuffciency for Znf9 in Znf9+/− mice is associated with multiorgan abnormalities resembling myotonic dystrophy J Mol Biol 2007 368 1 8 17 10.1016/j.jmb.2007.01.088 17335846

[B27] Aoki Y Niihori T Kawame H Kurosawa K Ohashi H Tanaka Y Filocamo M Kato K Suzuki Y Kure S Germline mutations in HRAS proto-oncogene cause Costello syndrome Nat Genet 2005 37 10 1038 1040 10.1038/ng1641 16170316

[B28] Deng Y Hu LS Lu GX Expression and identification of a novel apoptosis gene Spata17 (MSRG-11) in mouse spermatogenic cells Acta Biochim Biophys Sin (Shanghai) 2006 38 1 37 45 10.1111/j.1745-7270.2006.00125.x 16395525

[B29] da Huang W Sherman BT Lempicki RA Systematic and integrative analysis of large gene lists using DAVID bioinformatics resources Nat Protoc 2009 4 1 44 57 19131956 10.1038/nprot.2008.211

