# Supplementary figures and images for: Identification of proteins binding coding and non-coding human RNAs using protein microarrays
Source: BMC Genomics. 2012 Nov 16;13:633. doi: 10.1186/1471-2164-13-633 (PMC3562209; doi:10.1186/1471-2164-13-633)

## Figure S1

# A

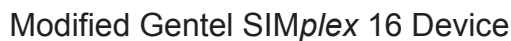

## Gasket

Spacer

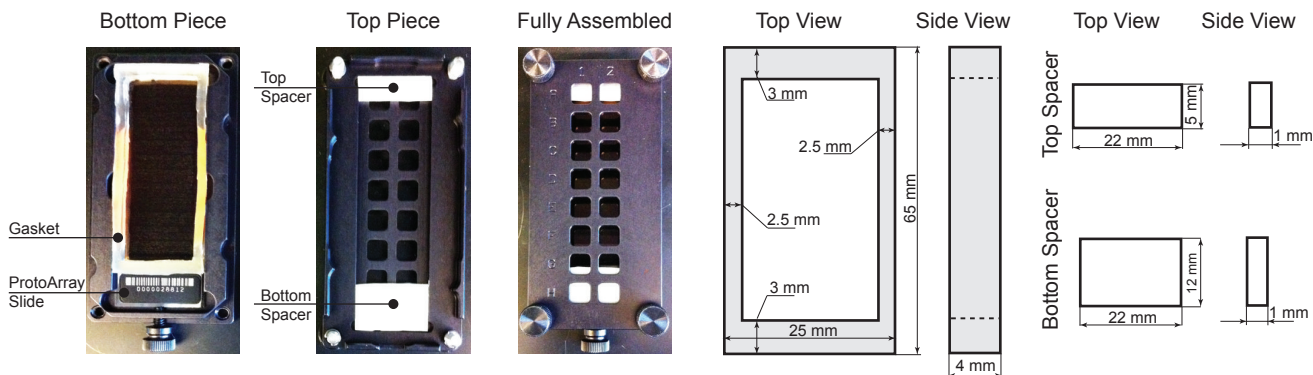

B

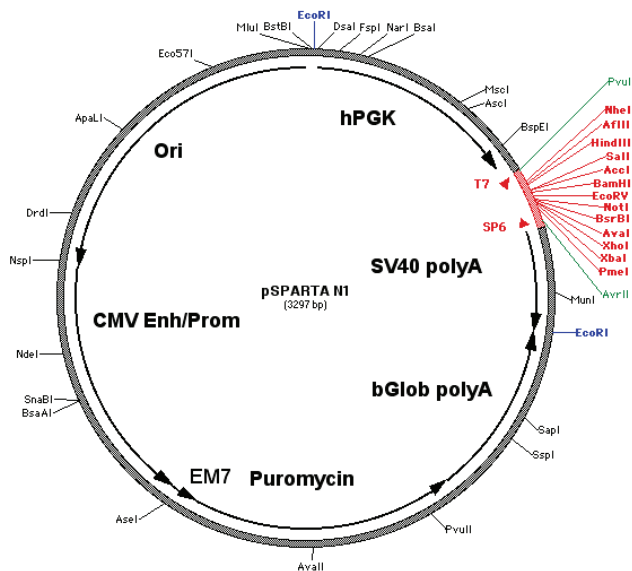

C

D

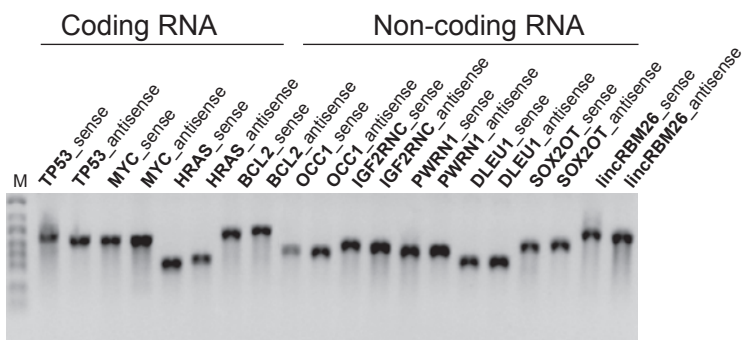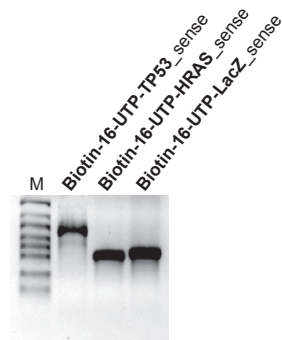

Supplement: Additional file 1 — Figure S1. Protein microarray incubation device and RNAs used for this work. (A) Modified Gentel SIMplex 16 device with microarray slide and assembly components. Schematic diagram of the custom-made silicone gasket and spacer with main dimensions indicated. (B) Expression vector pSPARTA. hPGK - human phosphoglycerate kinase promoter, SV40 polyA - simian virus 40 polyadenylation signal, bGlob polyA - beta-globin polyadenylation signal, Puromycin - resistance gene, EM7 - bacterial promoter, CMV Enh/Prom - cytomegalovirus enhancer promoter, Ori - origin of replication. Unique site depicted in black. Polylinker sites are in red. (C) Denaturing agarose gel electrophoresis of sense and antisense RNAs used in this work. M - RiboRuler RNA ladder (bp): 6000, 4000, 3000, 2000, 1500, 1000, 500, 200. (D) Denaturing agarose gel electrophoresis of biotin-16-UTP labeled RNAs TP53, HRAS, LacZ (1.2kb fragment of LacZ, experimental procedures). M - RiboRuler RNA ladder (bp): 6000, 4000, 3000, 2000, 1500, 1000, 500, 200. [file 1471-2164-13-633-S1.pdf]
