# Supplementary material for: Identification of proteins binding coding and non-coding human RNAs using protein microarrays
Source: BMC Genomics. 2012 Nov 16;13:633. doi: 10.1186/1471-2164-13-633 (PMC3562209; doi:10.1186/1471-2164-13-633)
Supplement: Additional file 4 — Figure S2. Confirmation of RNA-protein binding on microarrays. The reciprocal pull-down assays for CNBP with HRAS mRNA. (A) Quantitation images of human microarray showing selective binding signal of HRAS mRNA sense strand to duplicate CNBP protein spots. The incubation signal shown with respect to adjacent protein controls in the same sub-array. (B) Pull-down of biotin labeled HRAS mRNA in vitro, but not TP53 or LacZ precipitates associated HA-CNBP protein; densitometry quantification of the immunoblots shown (right). (C) HA-CNBP protein pulls down HRAS mRNA in vitro after immunoprecipitation with HA Mab; immunoblots to HA-tagged CNBP verifying CNBP precipitation are shown on the left panel. (D) HA-CNBP protein pulls down HRAS mRNA in vivo, but not control TP53 and GAPDH mRNAs after immunoprecipitation with HA Mab; immunoblots to HA-tagged CNBP verifying CNBP precipitation from cell extracts are shown (left). (E) Incubation of TP53-ORF mRNA sense strand lacking 5′ and 3′ UTR regions to human protein microarrays. Panel at left shows the entire microarray spotted with ~9400 recombinant human proteins; the middle panels represent an enlargement of the sub-array containing Stau1 and WIT1 proteins. Note absence of the Stau1-TP53 mRNA association signal in comparison to Figure 2D when full-length TP53 mRNA was probed. [all proteins spotted in duplicate; Stau1 and WIT1 spots boxed in white; sub-array positive controls boxed in red]. The quantification of the incubation results shown on the right. (F) Contrary to STAU1 depletion, UPF1 KD has no affect on TP53 RNA decay in Primary Fibroblasts. [file 1471-2164-13-633-S4.pdf]

Figure S2

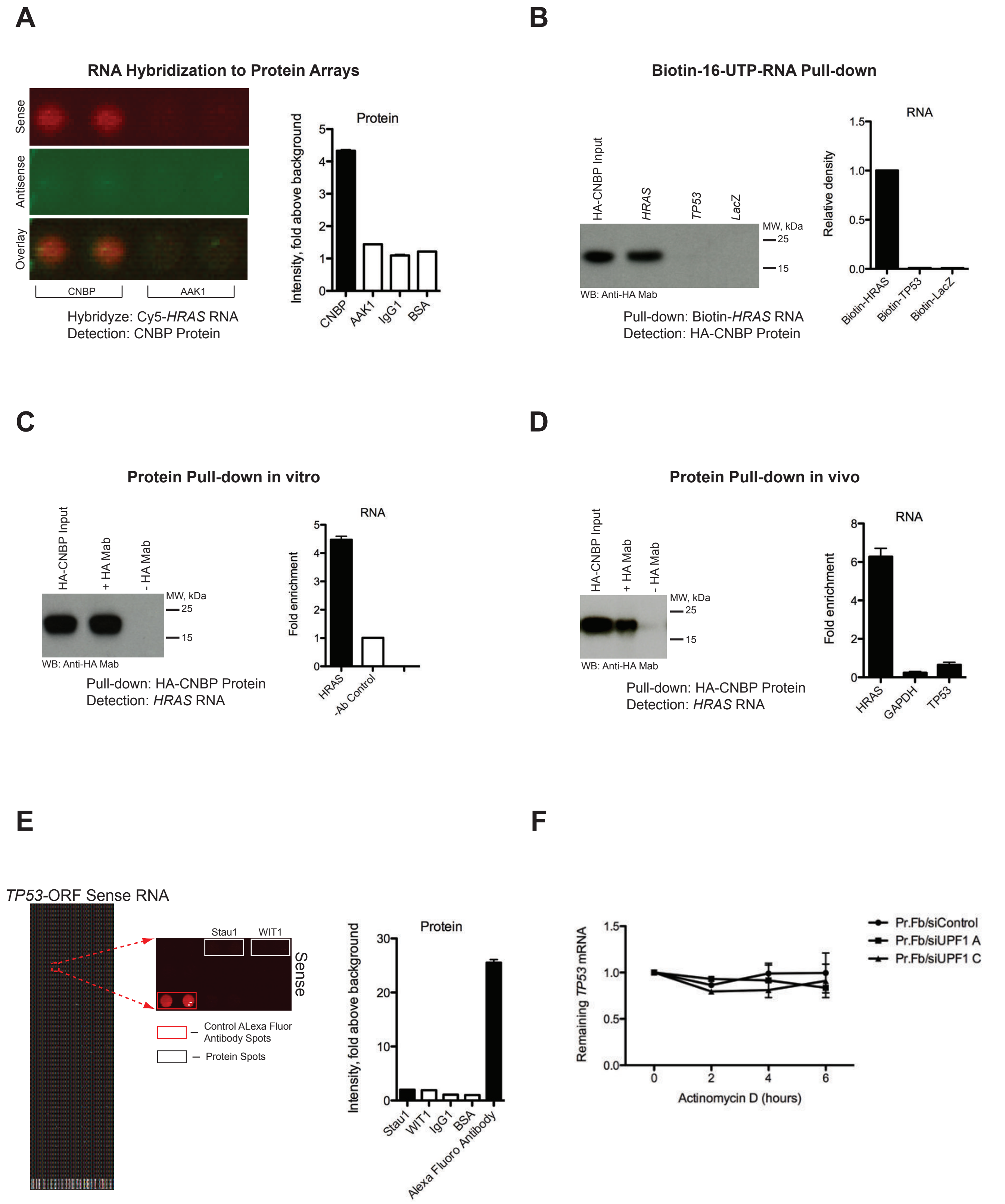
A
RNA Hybridization to Protein Arrays
Sense
Antisense
Overlay
CNBP
AAK1
Hybridyze: Cy5-HRAS RNA
Detection: CNBP Protein
Protein
Intensity, fold above background
5
4
3
2
1
0
CNBP
AAK1
IgG1
BSA
B
Biotin-16-UTP-RNA Pull-down
HA-CNBP Input
HRAS
TP53
LacZ
MW, kDa
25
15
WB: Anti-HA Mab
Pull-down: Biotin-HRAS RNA
Detection: HA-CNBP Protein
RNA
Relative density
1.5
1.0
0.5
0.0
Biotin-HRAS
Biotin-TP53
Biotin-LacZ
C
Protein Pull-down in vitro
HA-CNBP Input
+ HA Mab
- HA Mab
MW, kDa
25
15
WB: Anti-HA Mab
Pull-down: HA-CNBP Protein
Detection: HRAS RNA
RNA
Fold enrichment
5
4
3
2
1
0
HRAS
-Ab Control
D
Protein Pull-down in vivo
HA-CNBP Input
+ HA Mab
- HA Mab
MW, kDa
25
15
WB: Anti-HA Mab
Pull-down: HA-CNBP Protein
Detection: HRAS RNA
RNA
Fold enrichment
8
6
4
2
0
HRAS
GAPDH
TP53
E
TP53-ORF Sense RNA
Stau1
WIT1
Sense
Control ALexa Fluor Antibody Spots
Protein Spots
Protein
Intensity, fold above background
30
20
10
0
Stau1
WIT1
IgG1
BSA
Alexa Fluoro Antibody
F
Remaining TP53 mRNA
1.5
1.0
0.5
0.0
0
2
4
6
Actinomycin D (hours)
Pr.Fb/siControl
Pr.Fb/siUPF1 A
Pr.Fb/siUPF1 C
